# Anticholinergics and benzodiazepines on cognitive impairment among elderly with Alzheimer’s disease: a 1 year follow-up study

**DOI:** 10.1186/s13104-019-4874-z

**Published:** 2020-01-02

**Authors:** Rewadee Jenraumjit, Surarong Chinwong, Dujrudee Chinwong, Tipaporn Kanjanarach, Thanat Kshetradat, Tinakon Wongpakaran, Nahathai Wongpakaran

**Affiliations:** 1 Department of Pharmaceutical Care, Faculty of Pharmacy, Chiang Mai University, 239 Suthep Road, Muang Chiang Mai District, Chiang Mai, 50200 Thailand; 2 Department of Social and Administrative Pharmacy, Faculty of Pharmaceutical Sciences, Khon Kaen University, Khon Kaen, Thailand; 3 Department of Pharmacy, Maharaj Nakorn Chiang Mai Hospital, Chiang Mai, Thailand; 4 Geriatric Psychiatry Unit, Department of Psychiatry, Faculty of Medicine, Chiang Mai University, 110 Intawaroros Rd., T. Sriphum, A. Muang, Chiang Mai, 50200 Thailand

**Keywords:** Anticholinergics, Acetylcholine esterase inhibitors, Prescription, Alzheimer’s disease, Cognition

## Abstract

**Objective:**

Age-associated decline in central cholinergic activity makes older adults susceptible to harmful effects of anticholinergics (ACs). Evidence exists of an association between effects of AC medications on cognition. This retrospective cohort study examines how ACs affect cognition among older adults with Alzheimer’s disease (AD) who received acetylcholine esterase inhibitors (AChEIs) over the course of 12 months.

**Results:**

A total of 133 (80% women, mean age 78.38 years, SD 7.4) were recruited. No difference in sex, age and comorbid diseases was observed between participants who took ACs, benzodiazepines (BZDs) and AChEIs. The most common prescribed ACs was quetiapine, being used for behavioral and psychological symptoms (BPSD). Multilevel analysis showed that the change of mental state examination scores were significantly predicted in the group using ACs (*t* (169), − 2.52, p = .020) but not with the groups using BZD (*t* (162), 0.84, p = .440). Evidence showed that older adults with Alzheimer’s disease and exposed to ACs exhibited lower global cognitive scores than those without AC exposure. Using ACs could be a trade-off between controlling BPSD and aggravating cognitive impairment. Highlighting the awareness of the potential anticholinergic effect is important and may be the best policy.

## Introduction

Dementia is a common health problem among the elderly. In 2010, five millions of people in the US experience Alzheimer’s disease (AD) [1]. In Japan, the prevalence of dementia according to the DSM-IV or DSM-III-R criteria was 3.8% to 11.0% among peopled age 65 years old and older [2]. In Thailand, a national survey of 4048 elderly adults (age 60 and older) reported the prevalence of dementia was 3.3% [3]. Dementia prevalence among Thai elderly adults increased with age [4].

Alzheimer’s disease (AD) is the most common type of dementia. In patients with AD, the most destroyed neuronal cells are cholinergic neurons causing interruption in cholinergic transmission resulting in cognitive impairment [5–7]. Acetylcholine esterase inhibitors (AChEIs) help inhibiting cholinesterase enzyme, resulting in an increase of cholinergic transmission at the synapses. Longitudinal studies have shown that AChEI, particularly higher doses, produced better longitudinal cognitive outcomes [8, 9].

That anticholinergics (ACs) should not be recommended among the elderly is widely known [10–12], and, in particular, patients with dementia [13] because significant detrimental effects of ACs on cognition have been clearly demonstrated [14–16].

Despite the fact that robust evidence exists regarding how ACs can affect both cognition and AChEIs per se due to pharmacodynamics interactions, based on their opposing mechanisms of action [17], most were from cross-sectional and short term follow-up study. A recent 36-month follow-up research showed that high anticholinergic burden negatively affected the treatment response to cholinesterase inhibitors and that an average ACB score > 3 was an independent prognostic factor for delirium or mortality among dementia patients [18]. However, no clear relationships between ACs and the change of cognition were noted in this study.

In addition to ACs, benzodiazepines (BZD), is considered a harmful medication and recommended by the American Geriatric Society to avoid among adults beginning 65 years of age, particularly. It should also be avoided among older adults with dementia, delirium or cognitive impairment due to its worsening cognitive impairment and increased risks or delirium, falls, fractures and accidents [19]. However it can still found being used among geriatric patients [20].

To focus on using both ACs and/or BZDs among the elderly with dementia, related studies have revealed that a small number of studies investigating the effect of ACs on cognition in longitudinal design suggested a deleterious effect of anticholinergic exposure on long term cognitive function [21]. However, longitudinal studies investigating both ACs and BZDs on cognition with individuals with dementia receiving AChEIs are relatively few. To elucidate these relationships, the aim of this study was to investigate any association between ACs and BZDs on cognition among the elderly with AD receiving these medications and AChEIs in a longitudinal fashion, in addition to exploring the prevalence of using these medications among old aged patients with AD.

## Main text

### Methods

The study was approved by an independent Ethics Committee for Human Research, Faculty of Medicine, Chiang Mai University, Thailand. This was a longitudinal study conducted in 2018 by reviewing outpatient medical records of elderly Thai patients who had experienced AD and were followed up in a university hospital of northern Thailand from 2015 to 2016. The medical records were recruited by the Department of Medical Informatics to identify medical records with ICD-10 diagnoses of codes F00. The inclusion criteria for the present study included the patient who, (1) was 60 years old and above, (2) was diagnosed by physicians specialized in AD, (3) received AChEIs, (4) had a Thai mental status examination score (TMSE) [22, 23] regularly during each visit.

The baseline was determined when the patient received AChEIs and every 4 months (± 3 to 4 weeks), patients were assessed using the TMSE Data including demographic information, i.e., sex, age, underlying diseases etc., and the names of all oral medications prescribed in 1 year including AChEIs, ACs and BZDs.

#### Evaluating anticholinergic effects among medications

Anticholinergic effects were evaluated by the Anticholinergic Cognitive Burden (ACB) [24] which describes all possible anticholinergics including those listed with a score of 1; definite anticholinergics including those listed with a score of 2 or 3. An example of a drug in category ACB score 1 was diazepam, score 2 was cyproheptadine and score 3 was amitriptyline.

#### Statistical analysis

Descriptive data were analyzed in percentages, means and standard deviations (SD). The relationship between prescribing ACs, BZDs and comorbidities, was determined with the Chi-square test. The relationship between prescribing ACs and BZDs and the change of TMSE scores over 12 months was determined using a hierarchical linear model (multilevel analysis). Two-tailed tests were used to determine the statistical significance at p-value less than .05. The data were analyzed using IBM SPSS, Version 22.0 (SPSS Inc., Chicago, IL, USA), and longitudinal data analyzed using HLM, Version 8 (Scientific Software International, Inc., Chicago, IL, USA).

### Results

One hundred eighty-four medical records were analyzed. A total of 133 (80% women, mean age 78.38 years, SD 7.4) met the criteria for the study. Table 1 shows the demographic data, severity of dementia by TMSE scores and comorbid physical diseases. No statistical difference was found between the groups with or without ACs and the groups with or without BZDs according to age, sex, health privilege, ICD-10 diagnosis and comorbidities. TMSE scores significantly differed at baseline (time 1) between AC and nonAC group, unlike BZD groups. The difference of TMSE scores between groups were observed at time 3 and 4 (at months 8 and 12).

**Table 1:** Sociodemographic characteristics and clinical data of the sample

Characteristics	N (%) or mean (SD)	AC		BZD	
	All (n = 133)	Yes (n = 42)	No (n = 91)	Yes (n = 9)	No (n = 124)
Age (year)	78.38 (7.4) Range (60–94)	77.88 (8.04) Range (60–94)	78.60 (7.18) Range (63–94)	79.67 (7.12) Range 70–93	78.28 (7.48) Range (60–94)
Sex, female	80 (60.2)	28 (66.7)	52 (57.1)	6 (66.7)	74 (59.7)
Health privilege					
Universal coverage	1 (0.8)	1 (2.4)	0 (0.0)	0 (0.0)	1 (0.8)
Government	120 (90.2)	39 (92.9)	81 (89.0)	9 (100.0)	111 (89.5)
Social	1 (0.8)	0 (0)	1 (1.1)	0 (0.0)	1 (0.8)
Others	11 (8.3)	2 (4.8)	9 (9.9)	0 (0.0)	11 (8.9)
ICD-10 diagnosis					
F000	22 (16.5)	7 (16.7)	16 (17.6)	2 (22.2)	21 (16.9)
F001	96 (72.2)	31 (73.9)	72 (79.1)	6 (66.7)	97 (78.2)
Others	15 (11.3)	4 (9.4)	3 (3.3)	1 (11.1)	6 (4.9)
Drugs used					
Anticholinergics	42 (31.6)	–	–	–	–
Benzodiazepines	9 (6.8)	–	–	–	–
Comorbidity	116 (87.2)	41 (97.6)	75 (82.4)	8 (88.9)	108 (87.1)
Infection	2 (1.5)	1 (2.4)	1 (1.1)	0 (0.0)	2 (1.6)
Cancer	2 (1.5)	1 (2.4)	1 (1.1)	0 (0.0)	2 (1.6)
Hematology	5 (3.8)	2 (4.8)	3 (3.3)	0 (0.0)	5 (4.0)
Metabolic disease	68 (51.1)	22 (52.4)	46 (50.5)	4 (44.4)	64 (51.6)
Psychiatry	18 (13.5)	8 (19.0)	10 (11.0)	2 (22.2)	16 (12.9)
Neurology	30 (22.6)	12 (28.6)	18 (19.8)	0 (0.0)	30 (24.2)
Eyes and ears	3 (2.3)	1 (2.4)	2 (2.2)	0 (0.0)	3 (2.4)
Cardiovascular	87 (65.4)	28 (66.7)	59 (64.8)	5 (55.6)	82 (66.1)
Respiratory	3 (2.3)	2 (4.8)	1 (1.1)	1 (11.1)	2 (1.6)
Gastrointestinal	4 (3.0)	1 (2.4)	3 (3.3)	0 (0.0)	4 (3.2)
Skin	0 (0.0)	0 (0.0)	0 (0.0)	0 (0.0)	0 (0.0)
Musculoskeletal	23 (17.3)	14 (33.3)	9 (9.9)^†^	2 (22.2)	21 (16.9)
Genitourinary	16 (12.1)	4 (9.8)	12 (13.2)	0 (0.0)	16 (13.0)
TMSE score					
Visit 1	18.56 (6.71)	16.38 (7.39)	19.72 (6.71)**	15.80 (6.93)	19.01 (6.87)
Visit 2	18.97 (6.59)	17.21 (6.71)	19.49 (6.82)	17.67 (6.16)	18.78 (6.91)
Visit 3	20.06 (5.54)	18.26 (6.21)	21.45 (4.86)**	18.78 (6.91)	19.88 (4.55)
Visit 4	19.96 (6.74)	16.96 (7.08)	22.46 (5.69)**	14.50 (9.22)	20.80 (6.36)*

*AC* anticholinergic, *BZD* benzodiazepine, *SD* standard deviation, *ICD-10* International Classification of Disease—10th edition, *F000* dementia in Alzheimer’s disease with early onset, *F001* dementia in Alzheimer’s disease with late onset, *TMSE* Thai mental state examination

* p < .05, ** p < .01

^†^Significant difference between group (χ^2^(1) = 11.04, p = .002)

The prevalence of anticholinergic drugs prescription was 31.6%. The common ACs with an anticholinergic burden and doses are shown in Table 2. According to Table 2, the most frequent prescription among the anticholinergics with an ACB score of 3 was quetiapine while the most frequent for the anticholinergics with ACB score of 1 was aripiprazole. The other ACs prescribed included risperidone, cetirizine, clozapine, codeine, desloratadine, loratadine and olanzapine. For AChEIs, the most commonly prescribed was rivastigmine, while lorazepam was the most common for BDZ (see Additional file 1: Table S1). Only five (3.8%) of the patients received both ACs and BZD.

**Table 2:** Anticholinergic medications with an ACB score, and doses (n = 42)

Medicine	ACB score	N (%) of cases	Dose		N (%)
			Mean (SD), median	Dose (mg) per day	
Quetiapine	3	23 (54.8)	24.54 (14.21), 25.0	6.25	3 (7.1)
				12.50	13 (31.0)
				25.00	17 (40.5)
				50.00	8 (19.0)
Aripiprazole	1	7 (16.7)	3.75 (1.46), 5.0	1.25	2 (10)
				2.50	7 (35)
				5.00	11 (55)
Trazodone	1	3 (7.1)	62.5 (13.36), 62.50	50.00	4 (50.0)
				75.00	4 (50.0)
Hydroxyzine	3	2 (4.8)	3.67 (.75), 3.5	3.00	3 (50.0)
				4.00	1 (16.7)
				4.50	2 (33.3)
Others^a^	–	7 (16.6)	–	–	–

^a^Including risperidone, cetirizine, clozapine, codeine, desloratadine, loratadine, and olanzapine

The fixed effect from the hierarchical linear modeling showed that the anticholinergic group changed more significantly in the slope of TMSE than the nonanticholinergic group (b = − 2.519, 95% CI .399, 4.639) but not with benzodiazepine (b = − 1.662, 95% CI − 2.232, 5.557). Likewise, advancing age predicted a significantly negative slope of TMSE (b = − .193, 95% CI − .327, − .060). However, time was not a predictor of TMSE score (Table 3).

**Table 3:** Predictors for TMSE scores

Parameter	Estimate	Std. error	df	t	p-value	95% confidence interval	
						Lower bound	Upper bound
Intercept	29.93	5.80	130.06	5.16	.000	18.45	41.41
Time	0.17	0.16	273.54	1.08	.282	− 0.14	.47
Male	− 1.34	1.02	133.36	− 1.32	.191	− 0.67	3.34
Age	− 0.194	.07	135.61	− 2.88	.005	− 0.33	− 0.07
BDZs	− 1.66	1.97	127.16	0.84	.400	− 2.23	5.56
Anticholinergics	− 2.52	1.12	129.04	− 2.35	.020	0.400	4.64

*SD* standard deviation, *TMSE* Thai mental state examination, *BZD* benzodiazepine

### Discussion

To the best of our knowledge, this study was the first to report on the AC prescription among Thai patients with AD including the prevalence of AC prescription, the concomitant use with AChEIs and the association with adverse effects on cognition among elderly Thais with AD.

The authors found a high rate of AC drugs among patients with AD (31.6%) even though this was lower than that in other studies ranging from 46.83% to 65.8 [25, 26]. The reason could be that our participants were collected from tertiary care, a university hospital of northern Thailand. This group of patients had concomitant medications from other secondary care centers, in which we have not yet had an effective accessible system among hospitals and other health care providers. This gap may have allowed unnecessary and unthoughtful prescription of ACs to take place.

The present study showed adverse effects on cognition among the elderly with AD. TMSE score was lower with statistical significance among patients that were AC concomitant even though they received AChEIs. The study confirmed the negative predictors of TMSE score that could be predicted by advanced age and AC use. These were in line with a 2-year longitudinal study of the elderly in that the use of AC medication with definite anticholinergic effects was associated with a greater decline in MMSE score than not taking anticholinergics, whereas the use of possible anticholinergics at baseline was not associated with further decline [16]. Advancing age was, as expected, another predictor for longitudinal outcome of cognition, as found in related research [9]. Notably, not only did the anticholinergic effect have a direct impact on cognition, a pharmacodynamic drug interaction between AChEIs and AC also nullified the benefit of AChEI at the neuronal level. However, we are not yet able to conclude that any pair of combinations of AC and AChEIs has the same effect on cognition. Verifying this may require a large sample size. Despite that, clinicians should be more aware of using ACs among patients receiving AChEIs because it may not only worsen cognition but also become a huge loss regarding economic aspects.

One important point to be note is that the most common AC drug used was quetiapine, which is mostly related to remedy behavioral and psychological symptoms of dementia (BPSD). This finding was in line with related studies in that quetiapine was the most used drug among patients with dementia and was harmful to cognitive outcome [27, 28]. For what reason, quetiapine has become commonly prescribed for clinicians remains unclear and may not be an easy answer to find using this type of research design. Several atypical antipsychotic drugs can be used among patients with disturbed symptoms of dementia. Aripiprazole and risperidone may be better than quetiapine in terms of anticholinergic effects, but provide more risk of extrapyramidal side effects. In complicated situations of the patient, clinicians should individualize the assessment of safety risks against expected benefits when prescribing atypical antipsychotics. Therefore, it may be difficult to simply suggest not using quetiapine with this situation. Consistent with the recent network meta-analysis, the study revealed a trade-off between the effectiveness and safety of atypical antipsychotics in the treatment of BPSD and assures that a single most effective and safe treatment option does not exist [29].

Regarding BZD, the present study showed that only a small percent of the patients received a combination between both ACs and BZD, which was lower than a related study in a large population (approximately 6%) [30]. A recent cohort study suggested that ACs or BZDs could increase dementia risk at 10-year follow-up. By that an ACB score of 3 was found, but neither BZDs nor ACB score 1 or 2 medications was associated with dementia, particularly in those with good baseline cognitive function [31]. According to a related study, our result demonstrated that BZDs use was not associated with a negative predictor of TMSE score. This finding was also supported by a longitudinal study in that MMSE was not associated with BZD use in the models [32]. Considering a relatively small sample size, we cannot conclude that no long-term effect exists of BZD concerning cognition, on the contrary, prescribing long term BZDs among the elderly regardless concomitant with AC or no AC should be cautious, as it remains potentially inappropriate due to other harmful effects of BZD [33].

In conclusion, despite the fact that AC medication should be avoided among patients with dementia, it may not be easy to avoid using medication with anticholinergic effects especially atypical antipsychotic to deal with BPSD. AC drugs and age were the strong predictors of negative cognitive outcomes in the long run. Awareness of potential anticholinergic risk of medication seems to be the best policy. In a setting where a geriatric physician or nurse is lacking, the incidence of prescribing such medication is relative high. Therefore, maintaining awareness and monitoring whenever these anticholinergic drugs are used is important regardless of what use they are for.

## Limitations

The main limitation of the present study is the small sample size which may have an impact on statistical power. A larger sample is required, particularly for subgroup analysis of the combinatorial effects of AC and AChEIs on cognition. The method used made information regarding the patients accessed in other health care centers unavailable. In addition, over-the-counter medications were unreported. Moreover, being prescribed a medication does not mean that the patients have actually taken them. Finally, further prospective studies with larger populations should be warranted to demonstrate proportion and the impact of AC use.

## Supplementary information

**Additional file 1: Table S1.** AChEIs and BZDs.

## Data Availability

The datasets used and/or analyzed during the current study are available from the corresponding author upon reasonable request as data sharing is subject to the Ethics Office for approval.

## Abbreviations

AC: anticholinergic
ACB: Anticholinergic Cognitive Burden
AChEI: acetylcholine esterase inhibitor
AD: Alzheimer’s disease
ARS: Anticholinergic Risk Scale
BPSD: behavioral and psychological symptoms of dementia
BZD: benzodiazepine
TMSE: Thai mental status examination
