# Supplementary material for: Anticholinergics and benzodiazepines on cognitive impairment among elderly with Alzheimer’s disease: a 1 year follow-up study
Source: BMC Res Notes. 2020 Jan 2;13:4. doi: 10.1186/s13104-019-4874-z (PMC6941288; doi:10.1186/s13104-019-4874-z)
Supplement: Supplementary file 1 — Additional file 1: Table S1. AChEIs and BZDs. [file 13104_2019_4874_MOESM1_ESM.docx]

| **Table S1**. AChEIs and BZDs | | |
| --- | --- | --- |
| AChEIs ( n = 133) | n | percent |
| Donepezil | 37 | 27.8 |
| Galantamine | 24 | 18.0 |
| Rivastigmine | 46 | 34.6 |
| Memantine | 11 | 8.3 |
| Rivastigmine + Memantine | 7 | 5.3 |
| Donepezil + Memantine | 5 | 3.8 |
| Galantamine + Memantine | 3 | 2.3 |
| BDZs ( n =9) |  |  |
| Clonazepam | 2 | 22.2 |
| Lorazepam | 4 | 44.4 |
| Alprazolam | 2 | 22.2 |
| Zolpidem* | 1 | 11.1 |
| Note: AChEIs = Acetyl Cholinesterase Inhibitor, BZDs = Benzodiazepine  *Non-benzodiazepines (Z-drugs) | | |
